# Radioimmunotherapy in HPV-Associated Head and Neck Squamous Cell Carcinoma

**DOI:** 10.3390/biomedicines10081990

**Published:** 2022-08-17

**Authors:** Xin Zhou, Xiaoshen Wang

**Affiliations:** 1 Department of Radiation Oncology, Fudan University Shanghai Cancer Center, Shanghai 200032, China; 2 Department of Oncology, Shanghai Medical College, Fudan University, Shanghai 200032, China; 3 Shanghai Key Laboratory of Radiation Oncology, Shanghai 200032, China; 4 Department of Radiation Oncology, Eye & ENT Hospital, Fudan University, Shanghai 200032, China

**Keywords:** human papillomavirus, head and neck cancer, radiotherapy, immunotherapy

## Abstract

HPV-associated head and neck squamous cell carcinoma (HNSCC) is a cancer entity with unique biological and clinical characteristics that requires more personalized treatment strategies. As the backbone of conventional therapeutics, radiation is now harnessed to synergize with immunotherapy in multiple malignancies. Accumulating preclinical and clinical data have suggested the potential of radioimmunotherapy in eliciting local and systemic anti-tumor response via direct killing of tumor cells and immunogenic cell death. However, this effect remains uncertain in HPV-associated HNSCC. Owing to its intrinsic radiosensitivity and distinct tumor microenvironment, HPV-associated HNSCC may represent a good candidate for radioimmunotherapy. In this review, we provide a detailed illustration of the biology, the genomic features, and immune landscapes of HPV-associated HNSCC that support the synergism between radiation and immune agents. The interaction between radiotherapy and immunotherapy is described. We also highlight the present evidence as well as ongoing trials using different combination strategies in the recurrent/metastatic or definitive settings. In addition, we have summarized the challenges and outlook for future trial design, with special emphasis on radiotherapy optimization and novel therapeutic options to incorporate.

## 1. Introduction

With the global prevalence of human papillomavirus (HPV) as a most common sexually transmitted infection, the incidence of HPV-associated head and neck squamous cell carcinoma (HNSCC), particularly oropharyngeal carcinoma (OPC), has dramatically increased over the past decades. Currently, HPV-associated OPC accounts for 33% of all cases globally, with a highest prevalence reported in Lebanon (85%) and Sweden (70%) [1,2]. Radiotherapy has been the mainstay of conventional treatment for HPV-associated HNSCC for a long time, either to provide locoregional tumor control in a definitive setting or to yield palliative ablation in distant metastases [3]. Over the past decade, immune checkpoint inhibitors (ICIs) against cytotoxic T-lymphocyte-associated protein 4 (CTLA-4), programmed cell death protein 1 (PD-1) and programmed death-ligand 1 (PD-L1) have revolutionized the treatment of HNSCC including HPV-associated cancers with impressive anti-tumor activity and survival outcomes. Second-line treatment with ICI alone in recurrent and/or metastatic HNSCC reduced the risk of death by 20–30% with an overall response rate (ORR) of 13–15%, while ICI plus chemotherapy significantly improved overall survival compared with standard EXTREME regimen in the first-line setting [4,5,6]. However, ICIs alone are usually too limited to trigger a durable strong immune response. Growing interests are focusing on the use of radiotherapy (RT) to augment the effect of ICIs, thus leading to a new era of radioimmunotherapy. In this review, we will summarize the preclinical and clinical data of radioimmunotherapy in HPV-associated HNSCC on the basis of HPV-driven mechanisms, including the genetic and immunologic alterations, as well as the immunomodulatory effect of RT on tumor microenvironment.

## 2. Clinical Presentations and Biology of HPV-Associated HNSCC

Clinically, HPV-associated HNSCC is featured by a proclivity towards male, younger age of onset, less consumption of tobacco and alcohol, specific anatomic site in oropharynx, and a histology of non-keratinized squamous cell carcinoma [7]. As a distinct subset of HNSCC, HPV-associated cancers are well acknowledged with better prognosis compared to their HPV-negative counterparts [8,9], which further led to a separate classification in the 8th edition of American Joint Committee on Cancer (AJCC) staging system. This favorable outcome was conceivably attributed to higher intrinsic sensitivity to radiation and chemotherapy [8,10], which prompted a plethora of strategies on treatment de-intensification to expand the therapeutic ratio in HPV-associated HNSCC.

**Figure 1:**
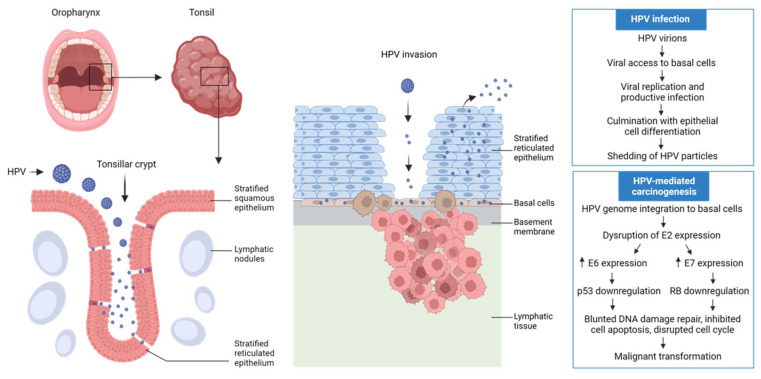
HPV infection and carcinogenesis of HPV-associated oropharyngeal carcinoma. Tonsillar crypts are lined with stratified reticulated epithelium which is characterized by discontinuous basal lamina and incomplete differentiation. This disrupted nature enables HPV to access the basal cells and the basement membrane, forming a productive infection status through active replication of mature HPV virions which eventually culminate and shed with the differentiation of epithelial cells. High-risk subtypes of HPV can stably integrate into the host genome with their DNA, followed by a downregulation of E2 expression and upregulation of E6 and E7 expression, which further disrupt the intracellular p53 and RB levels respectively. The p53-mediated cell apoptosis and DNA damage repair, as well as RB-mediated cell cycle regulation are then crippled, leading to malignant transformation and subsequent tumor invasion. This figure was created with BioRender.com.

High-risk HPV strains, predominantly HPV16, represent the major driving factors that mediate the oncogenesis of HPV-associated HNSCC. Despite lacking direct evidence of precancerous lesions induced by HPV infection, HPV-associated HNSCC possibly resembles HPV-driven ano-genital cancers in their development that involves progression from normal epithelium to dysplasia, carcinoma in situ and eventually invasive cancer [11,12]. In line with the predilection of onset in lingual or palatine tonsils, HPV16 preferentially targets the reticulated epithelium in tonsillar crypts to induce a persistent infection status. With the integration of single or multiple copies of viral genome into host chromosomes [13], the viral gene E2 undergoes a loss of transcript production, which in turn upregulates the transcription of E6 and E7. The E6 protein induces the degradation of tumor-suppressor P53 through ubiquitin-mediated proteolysis, blunts P53-mediated cell apoptosis and repair of DNA damage, thus rendering host cells more susceptible to genomic instability [14]. The E7 protein, through binding and inactivating pRb, releases the E2F family of transcription factors and disrupts the cell-cycle by driving cells into the S-phase, eventually leading to abnormal cell proliferation and malignant transformation [15] (Figure 1). Besides these canonical mechanisms, E6 and E7 oncoproteins also exert pleiotropic functions involving epigenome disruption [16], cell immortalization [17] as well as innate immune response [18].

## 3. Genomic and Immune Landscape of HPV-Associated HNSCC

Due to the common smoking history, HNSCC presents a relatively high tumor mutational burden (TMB) that ranks in the upper quartile among different cancer types [19]. According to Samstein et al., over 20% of HNSCC showed a TMB of ≥10 mut/Mb [20]. When regarding HPV status, HPV-positive HNSCC showed similar TMB with HPV-negative cancers, whereas with different mutational landscape [21], generally, HPV-positive HNSCC exhibits rarer KRAS mutations and higher frequency of PIK3ca mutations that target the PI3K-AKTmTOR (PI3K) pathway; in particular, characteristic mutations including DDX3X, CYLD, and FGFR were also seen in HPV-positive HNSCC. Interestingly, despite the hypothesis that HPV-positive HNSCC might generate more neoantigens from viral integration, data from The Cancer Genome Atlas (TCGA) actually showed no significant differences in neoantigen count between HPV-positive and HPV-negative tumors [22], suggesting that they had different mechanisms in mediating tumor immunogenicity. 

HPV-associated HNSCC, primarily OPC, also exhibits unique immune microenvironment that harbors more pro-immunological features. Compared to its HPV-negative counterparts, HPV-positive HNSCC displays a T-cell-inflamed phenotype with increased CD4+ T-cells, CD8+ T-cells and cytokine production, as well as higher diversity of T-cell receptor repertoire [22,23,24]. The FoxP3 + Tregs are also enriched with tumor progression. However, HPV-positive HNSCC retains a higher ratio of total T-cells to Tregs than HPV-negative cancer, which is predictive of better survival outcomes possibly owing to an outweighing pro-immunity function over immunosuppression in tumor microenvironment that favors anti-cancer activity [25,26]. Significant increase of intra-tumoral myeloid dendritic cells is also found, suggesting a great potential of antigen recognition and presentation [27]. In addition, the HPV-associated HNSCC features enhanced pro-inflammatory innate immune response with more M1 macrophages (classically activated, tumoricidal) and less M0 (non-polarized, uncommitted) and M2 macrophages (alternately activated, tumor growth-promoting) [24], resulting in an increased M1/M2 ratio that favors better prognosis in this subset of patients [28]. Moreover, neutrophils that negatively impact survivals tend to decrease in HPV-associated HNSCC [29], partially due to the inhibitory function of E7 protein. 

Following T-cell activation, HPV-associated HNSCC develops a negative feedback loop to forge an immunosuppressive microenvironment with upregulated expression of CTLA4 [23], CD39 and multiple T-cell exhaustion markers including LAG3, PD1, TIGIT and TIM3 [24], as per transcriptome data from TCGA. Of note, these markers provide important targets for immunotherapy to prolong the activation status of T-cells and to promote the anti-tumor activity.

## 4. Sensitivity to Immunotherapy in HPV-Associated HNSCC

As discussed above, the genomic and immunologic features of HPV-associated HNSCC support the rationale of immunomodulatory agents, ICIs that block PD-1, PD-L1 or CTLA-4 in this population of patients. Given the pre-existing tumor-specific immune cells in HPV-positive tissues, the ICIs can effectively reverse the immunosuppressive microenvironment by releasing the “brake” and reactivating effector T-cells, thus reinvigorating the anti-tumor response. 

Although there is yet no direct comparison on the response to ICIs based on HPV status, a body of evidence has indicated a trend towards higher sensitivity in HPV-positive HNSCC. In the phase 1b KEYNOTE-012? trial, recurrent or metastatic HPV-positive HNSCC showed a higher ORR to pembrolizumab alone as first-line or subsequent-line treatment, when compared with its HPV-negative counterpart (25% vs. 14%) [30]. Ensuing data from an expansion cohort of KEYNOTE-012 showed a larger ORR benefit (32% vs. 14%), which further led to better 6-month progression-free survival (PFS) (37% vs. 20%) and overall survival (OS) (70% vs. 56%) in HPV-positive patients [31]. Similar effects of ORR were reported with Nivolumab (HPV-positive vs. HPV-negative, 15.9% vs. 8.0%) [5] and annti-PD-L1 agent Durvalumab (HPV-positive vs. HPV-negative, 30% vs. 10.8%) [32] in recurrent or metastatic HNSCC. A pooled analysis from six prospective clinical trials of 589 patients with recurrent or metastatic HNSCC receiving ICIs also revealed a higher ORR in HPV-positive HNSCC irrespective of PD-L1 expression [21.9% vs. 14.1%, odds ratio (OR) = 1.79; *p* = 0.01] [22]. When applied in a neoadjuvant setting prior to surgery, nivolumab yielded a much higher pathologic regressions in HPV-positive than in HPV-negative patients (23.5% vs. 5.9%), as was found in the CheckMate 358 trial [33]. These data collectively suggest that HPV-positive patients might benefit more from ICIs owing to a higher immunotherapeutic sensitivity. The specific use of immunotherapy in HPV-positive HNSCC has been reviewed elsewhere and will not be discussed in this review [34,35,36].

## 5. Rationale for Combined Radiotherapy and Immunotherapy in HPV-Associated HNSCC

HPV-associated HNSCC is generally believed to be more sensitive to conventional chemotherapy and radiotherapy (RT). Direct evidence came from a trial by Fakhry et al., in which patients with HPV-positive HNSCC had higher ORR to induction chemotherapy (82% vs. 55%, *p* = 0.01) and chemoradiation (84% vs. 57%, *p* = 0.007) when compared to HPV-negative disease [8]. This effect was further determined in vitro and in vivo, where HPV-positive HNSCC exhibited superior intrinsic radiosensitivity mediated by HPV-derived oncoproteins [37]. The mechanisms for chemo-/radio- sensitivity in HPV-positive HNSCC involve impaired DNA damage repair pathways, increased G2/M arrest following radiation, a smaller population of cancer stem cells, dampened angiogenesis, and a less hypoxic microenvironment, as well as the expression of functional albeit low level wild-type p53 that plays a central role in inducing post-radiation apoptosis and tumor cell death [38,39,40]. 

Strikingly, radiosensitivity highly depends on immunity in cancers. Radiotherapy alone was not sufficient to eradicate tumors in vivo until adoptive transfer of antigen-specific cytotoxic T lymphocytes (CTLs) [41]. In biopsy specimens from colorectal cancer patients, better response to neoadjuvant chemoradiotherapy (CRT) was associated with high infiltration of CD8+ and CD4+ T-cells that reflect an active anti-tumor immunity, as well as decreased myeloid-derived suppressor cells [42]. Gene expression signatures from a pan-cancer database revealed a strong correlation between radiosensitivity index and immune activation [43] featured by an enrichment of interferon-associated signaling pathways and infiltration of active immune cell such as CD8+ T-cells, activated natural killer cells and M1 macrophages [44]. In vivo responses to radiotherapy were observed with much higher sensitivity in HPV-positive HNSCC than in HPV-negative mice; however, this effect existed only in immunocompetent mice but not in immunodeficient mice, suggesting that intact immune system plays a crucial role in the radiosensitivity of HPV-positive HNSCC [45]. Given the clinical findings of active immune response and accompanying T-cell exhaustion in HPV-positive HNSCC [46,47], immunotherapy with ICIs could hopefully restore the anti-tumor immunity, thus further augment the RT response as a radiosensitizer [48]. 

**Figure 2:**
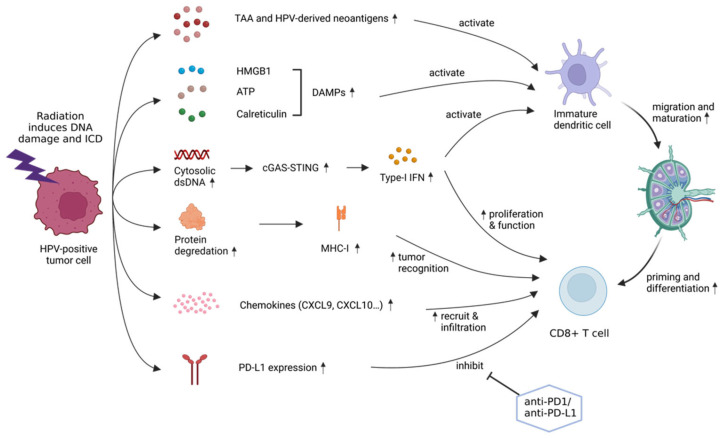
Immunomodulatory effects of radiotherapy in HPV-associated head and neck squamous cell carcinoma. This figure was created with BioRender.com.

Conversely, radiation can exert strong immunomodulatory effects on tumor microenvironment, which is regarded as the 6th “R” in radiobiology [49]. Multiple mechanisms of this effect have been illustrated: (1) radiation-induced cell death via DNA damage facilitates the release of tumor-associated antigens and neoantigens [50], causing an effect of “in situ vaccination” [51]; (2) radiation stimulates immunogenic cell death (ICD) via damage-associated molecular patterns (DAMP) such as calreticulin, adenosine triphosphate (ATP) and high-mobility group protein B1 (HMGB1) [52,53], which enhances the activation and migration of dendritic cells that are necessary for antigen presentation and T-cell priming; (3) radiation exposure increases cytosolic DNA, which upregulate the production of type-I IFN via cGMP-AMP (cyclic guanosine monophosphate-adenosine monophosphate) synthase-stimulator of IFN genes (cGAS-STING) DNA-sensing pathway [54,55]; (4) radiation upregulates the expression of MHC-I in tumor cells, thus enhancing the cross presentation of antigens [56]; (5) radiation upregulates chemokines such as CXC motif ligand 9 (CXCL9), CXCL10, CXCL11 and CXCL16, leading to increased recruitment of activated CD8+ T-cells [57,58]. In HPV-positive HNSCC, the cGAS-STING pathway is dampened by E7 with a reduction in type-I IFN response [59,60], which might be compensated with the combination of radiation. In addition, viral oncoproteins E6 and E7 provide neoantigens that favors an upregulated radiation-related immune response. Moreover, PD-L1 can be significantly upregulated after radiotherapy in HPV-associated OPC to antagonize anti-tumor activity, which can be reversed by anti-PD-1 or anti-PD-L1 antibodies [61] (Figure 2).

Taken together, the interplay of the immune response and radiotherapy warrants the application of radioimmunotherapy in malignant tumors, even those traditionally deemed with a “cold” tumor microenvironment. HPV-positive HNSCC, with its natural advantages in radiosensitivity and immune-sensitivity, might represent a good candidate for radioimmunotherapy to maximize the therapeutic ratio.

## 6. Radioimmunotherapy in Recurrent/Metastatic HPV-Associated HNSCC

Given its compelling immunomodulatory effects, radiotherapy has been applied in a metastatic setting in multiple malignancies to elicit systemic immune response against tumor, or putative “abscopal effect”, while attaining proper local control. As was described by R.H. Mole in 1953, an abscopal effect refers to a phenomenon whereby localized RT induced a tumor shrinkage in distant sites that were not exposed to irradiation [62]. However, this phenomenon remains extremely rare in sporadic cases until the advent of immunotherapy [63], which magnifies the prerequisites for abscopal effect, namely, an effective T-cell priming process and a CTL permissive tumor microenvironment [64]. So far, the strongest evidence of abscopal effect came from PEMBRO-RT (NCT02492568), a phase II randomized trial in NSCLC. By administering stereotactic body radiation therapy (SBRT) (8 Gy for 3 doses) prior to pembrolizumab, this trial resulted in a doubling of ORR at 12 weeks for non-irradiated lesions in the experimental arm when compared with patients receiving pembrolizumab alone (36% vs. 18%) [65]. Although it failed to reach the predefined primary endpoint to improve 12-week ORR from 20% to 50%, PEMBRO-RT did warrant more efforts to explore similar effects in other cancer species.

In HNSCC, however, a phase II trial (NCT02684253) from Memorial Sloan Kettering (MSK) Cancer Center failed to replicate the ORR advantage with the addition of single-lesion SBRT (9 Gy for 3 doses) to nivolumab (ORR, 29% in the experimental arm vs. 34.5% in the control arm, *p* = 0.86). When regarding the virus status of HPV or Epstein–Barr virus (EBV), subgroup analyses in the SBRT plus nivolumab arm found a numerically but not statistically superior response in virus-negative patients compared to that in virus-positive cases (26.7% vs. 14.2%, *p* = 0.16) [66]. These results in conflict with PEMBRO-RT and preclinical findings suggested the need to further elucidate the complex interplay of virus status, inflamed tumor microenvironment, ICIs, and ablative radiation in HNSCC. Of note, the relatively small sample size, timing of ICIs and potential impact of confounders such as heterogeneity in metastatic patterns and tumor burden may also influence the robustness of the conclusions in this study. Another phase II trial NCT04830267 is active enrolling to test the efficacy of SBRT plus camrelizumab in recurrent/metastatic HNSCC including HPV-positive patients. This study, with a priming phase of ICI prior to SBRT, will further shed light on the abscopal effect in HNSCC. Trials involving radioimmunotherapy in recurrent/metastatic HPV-associated HNSCC are summarized in Table 1.

## 7. Radioimmunotherapy in the Definitive Setting for HPV-Associated HNSCC

Although generally recognized with favorable prognosis, non-metastatic HPV-associated HNSCC is in fact a heterogenous entity with wide diversity of therapeutic effect and survival risk [67,68]. As mounting trials on the backbone of CRT have focused on treatment de-escalation to reduce toxicities including radiation-related dysphagia, mucositis, xerostomia and chemotherapy-induced hematological, gastrointestinal and neurological adverse events, successful experience was mostly based on specific selection criteria such as baseline tumor burden, smoking history [69], anatomic sites, response to induction chemotherapy [70] and post-operative pathological risk [71], while failures have been observed in unselected locoregionally-advanced patients [72,73]. These results highlighted the importance of patient selection for personalized intensity of treatment. Now in the new era of immunotherapy, ongoing trials in HPV-associated HNSCC with combined radiotherapy and immunotherapy in a definitive setting mainly involve two directions: (1) treatment escalation in intermediate-high risk patients; (2) treatment de-escalation in moderate-low risk patients. Relevant prospective trials are summarized in Table 2.

### 7.1. Addition of Concurrent ICIs to CRT

The JAVELIN Head and Neck 100 trial (NCT02952586) was the first randomized phase 3 study to combine an ICI with CRT in high-risk locoregionally-advanced HNSCC (LA-HNSCC). With the inclusion of HPV-positive OPC (T4, N2c-N3) and HPV-positive non-OPC, as well as HPV-negative HNSCC (stage III-IVb), this trial was designed to evaluate the PFS benefit of concurrent and adjuvant avelumab based on standard CRT. However, the trial was terminated early due to the failure to reach its primary endpoint. Although with fair tolerability, avelumab exerted no improvement in PFS or OS, either in the whole population, the HPV-positive or HPV-negative subgroups. A non-significant trend towards higher PFS was observed only in those with a high PD-L1 expression [74]. Compared to its successful counterpart, the PACIFIC trial in locally advanced non-small cell lung cancer (NSCLC) that incorporated PD-L1 blockade as consolidation treatment after CRT [75], JAVELIN 100 has aroused massive discussions on its failure. Plausible explanations would include the immunosuppressive tumor microenvironment in HNSCC, the depletion of effector T-cells due to a larger-volume elective irradiation and a different study design (PACIFIC study used no current ICIs). Furthermore, whether replacing anti-PD-L1 with anti-PD-1 antibodies will change the game remains unknown. 

A phase Ib trial (NCT02586207) reported the safety and efficacy of concurrent and adjuvant pembrolizumab in conjunction to CRT [76] in LA-HNSCC. The HPV-positive cohort had a higher completion rate of pembrolizumab (88.2% vs. 60%, *p* = 0.0118) and satisfactory outcomes (2-year OS 97.1%, 2-year PFS 92.8%). KEYNOTE-412 expanded this design into a phase III randomized trial. With enrollment of LA-HNSCC including p16-positive OPC (T4 or N3), KEYNOTE-412 was intended to answer the question whether escalating treatment intensity by adding pembrolizumab to CRT will translate into survival benefits eventually. The preliminary results will be released later in 2022.

### 7.2. Addition of Adjuvant ICIs to CRT

The ECOG ACRIN EA3161 (NCT03811015) is a phase II/III trial that aimed to investigate the survival benefit of maintenance nivolumab following definitive chemoradiation in PFS and OS amongst high-risk HPV-positive OPC, including those with more than 10 pack-years of smoking history and advanced stage of T1-2N2-N3 or T3-4N0-3. Another phase III trial, IMvoke010 (NCT03452137), randomized high-risk LA-HNSCC including stage III HPV-positive OPC to receive adjuvant atezolizumab after local radiotherapy or surgery. As the patient population in both trials features a high tendency of local progression and distant relapse, the addition of adjuvant ICI might hopefully help eradicate residual or micro-metastatic cancer clones.

### 7.3. Addition of Neoadjuvant ICIs to CRT

The IMMUNEBOOST (NCT03838263) trial introduced two cycles of neoadjuvant nivolumab to CRTs in high-risk HPV-positive OPC, also selected based on tumor stage (T4, N2/N3) and smoking history (>10 pack-years). The primary endpoint is the completion rate of full treatment. Interim analysis presented at the 2021 ASCO meeting showed the feasibility of this protocol, and the trial is continuing [77], awaiting more survival data in future. CompARE (NCT04116047) is a three-arm phase III trial to test alternative intensification of treatment in OPC with intermediate-high risk, including HPV-positive patients (N2b-N3, currently smoking or ≥10 pack years smoking history). After randomization, the experimental arms received either dose-escalated CRT or durvalumab prior to and following standard CRT. The NCT03829722 trial added nivolumab to a CRT protocol that adopted carboplatin/paclitaxel as concurrent chemotherapy. The ICI was used across the neoadjuvant, concurrent and adjuvant course. Another NCT05366166 trial, incorporated combined pembrolizumab and olaparib, a poly (adenosine diphosphate-ribose) polymerase (PARP) inhibitor, in the neoadjuvant (olaparib 150 mg twice daily for three weeks prior to chemoradiotherapy) and adjuvant phase (olaparib 150 mg twice daily for up to 48 weeks after chemoradiotherapy) to test the improvement of PFS in LA-HNSCC including HPV-positive patients. This trial will be initiated in June 2022.

### 7.4. Radioimmunotherapy-Based Treatment de-Escalation

A series of clinical trials assessed the feasibility of replacing concurrent standard-of-care agents with ICIs during radiotherapy in HPV-associated HNSCC. Single-agent ICI was used in the PembroRad trial, KEYCHAIN trial (NCT03383094), NRG-HN004 (NCT03258554), NRG-HN005 (NCT03952585) and CITHARE trial (NCT03623646) with similar design. The PembroRad trial has completed enrollment and the preliminary results were reported at the ESMO 2020 annual meeting. After enrolling 60% of patients with OPC (46% p16+), this trial found RT-pembrolizumab with less grade ≥ 3 acute toxicities but disappointingly no outcome improvement over RT-cetuximab [78], which has previously been proven inferior to standard CRT in PFS [72]. Whether RT-ICI could prove its efficacy in direct comparison to CRT in HPV-associated HNSCC still awaits results from KEYCHAIN and HN005 in future. 

The REACH study (NCT02999087) compared dual-agents of avelumab and cetuximab with cisplatin (100 mg/m² on days 1, 22, 43 concurrently with RT) in cisplatin-fit cohort and with cetuximab in cisplatin-unfit cohort among LA-HNSCC patients receiving definitive RT. With the inclusion of 61% cases with OPC (35% p16+), the REACH study showed a significantly lower rate of distant failure with RT-avelumab-cetuximab in the cisplatin-unfit cohort, suggesting the effect of ICI in eliminating potential micro-metastasis [79]. NCT03162731 is a phase I pilot study that evaluated dual-ICIs with nivolumab and ipilimumab before, with and after definitive RT in LA-HNSCC including HPV-associated diseases. Despite a 100% loco regional control, unexpectedly high in-field ulcerations were observed in 5 out of 24 patients within 3 months following RT [80], along with two cases developing adjacent necrosis, two with osteoradionecrosis and another two with persistent inflammation [81]. Though not determined, the combination of PD-1 and CTLA-4 blockade is highly suspected to cause these toxicities, and special caution should be taken in future studies when considering a dual-agent ICIs with RT. Another phase II trial using ipilimumab plus nivolumab is currently ongoing in advanced HPV-positive OPC, albeit with reduced-dose RT. Whether this combination could offset severe post-RT toxicities remains to be answered in the final results.

The NCT03715946 trial was designed to de-escalate the standard post-operative CRT by introducing ICI in p16-positive OPC with intermediate-high risk. Using concurrent and adjuvant nivolumab, the investigators expected to safely omit concurrent cisplatin and meanwhile reduce the RT dose to 45–50 Gy in 25 daily fractions.

Another important scenario for radioimmunotherapy-based de-escalation would be the combination of ICIs and induction chemotherapy to select patients for subsequent de-intensified RT. The OPTIMA-II (NCT03107182) is a phase II trial proposed on the heels of OPTIMA, which reported favorable outcomes and reduced toxicities in HPV-positive OPC, based on induction chemotherapy with carboplatin/nab-paclitaxel and ensuing de-intensified RT [82]. OPTIMA-II added nivolumab to the chemotherapy with the expectation of increased deep response rate (≥50% shrinkage of tumor to induction) and more patients amenable to response-adapted RT de-escalation [83]. The CheckRad-CD8 (NCT03426657) trial, after conducting one cycle of induction chemotherapy plus durvalumab and tremelimumab, selected patients with increased intra-tumoral density of CD8+ cells or pCR in re-biopsy to receive chemo-free radioimmunotherapy. Along with a demonstrated tolerability, the study found that patients with HPV-positive OPC benefit more from radioimmunotherapy with a 2-year PFS of 94% compared with 64% for HPV-negative OPC and non-OPC cancers [84]. The IChoice-02 (NCT04867330) study incorporated induction chemotherapy with toripalimab, an anti-PD-1 agent that has shown great efficacy in the first-line treatment of recurrent or metastatic nasopharyngeal carcinoma [85], to select HPV-positive OPC patients for de-escalated treatment with reduced-dose RT (60 Gy) and omitted concurrent cisplatin, also based on the deep response rate to induction chemoimmunotherapy.

### 7.5. Neoadjuvant Radioimmunotherapy Prior to Surgery

When applied prior to surgery, ICI alone is limited to induce satisfactory complete (pCR) or major (MPR) pathologic response in HNSCC including HPV-associated diseases [33,86]. Recent studies have focused on combining SBRT with ICIs in a neoadjuvant setting. The NIRT-HNC (NCT03247712) trial was the first phase Ib trial to test the efficacy of SBRT (40 Gy in 5 fractions or 24 Gy in 3 fractions) plus nivolumab given before definitive surgical resection in patients with LA-HNSCC. As most enrolled patients were HPV-positive (16/21, 76%), SBRT and nivolumab conferred a pCR advantage up to 90%, while only 50% of those receiving SBRT alone achieved pCR [87]. Mature data in future are needed to testify if the higher pCR will translate into survival prolongation, and to identify the individuals most likely to benefit from this combination. Another trial, NCT03618134, is also exploring radioimmunotherapy using SBRT plus durvalumab with or without Tremelimumab in HPV-positive OPC prior to transoral robotic surgery, further data are still awaited.

## 8. Challenges and Novel Concepts in Radioimmunotherapy

### 8.1. Radiation Dose and Fractionation

In general, radiation synergize with immunotherapy largely in a dose-dependent manner. Ablative RT, typically given with fractional dose >10 Gy for 1 to 5 fractions, mainly provides local tumor control via ICD-mediated mechanisms [56]. However, radiation dose escalated to 12–18 Gy would induce Trex1, a DNA exonuclease that abrogates the activation of cGAS/STING pathway through elimination of cytosolic DNA [88]. In comparison, SBRT with a sub-ablative fractional dose of 5–10 Gy may act as a stronger immunomodulatory regimen by increasing the release of chemokines and maintaining the cGAS/STING-mediated production of type-I IFN, thus boosting the infiltration of effector T-cells and anti-tumor immunity [89]. As preclinical studies using anti-CTLA-4 antibody in breast cancer and colon cancer mouse models observed an abscopal effect only when combining 8 Gy × 3 irradiation this fractionation has widely been used in clinical trials ever since. Low-dose (LD) irradiation with lower than 2 Gy, in contrast to ablative stereotactic radio surgery (SRS) and immunomodulatory SBRT, mainly impact on the tumor microenvironment through normalization of the tumor vascularity [90], better infiltration of immune cells, down-regulation of TGF-β [91] and polarization of macrophages to the M1 phenotype [92]. 

The fundamentally differing effect of high-dose and low dose- RT on immune response has led to the development of a new technique of “RadScopal” that integrates high-dose RT to generate “in situ vaccination” and low-dose RT to reverse stroma immunosuppression [93] and thus augment the abscopal effect. This strategy in combination with immunotherapy has shown great potential of anti-tumor response in prospective trials or post-hoc analysis of ongoing radioimmunotherapy trials, where low-dose RT yielded superior ORR than no-dose in secondary metastatic lesions among patients receiving high-dose RT plus ICIs [94,95,96]. As this approach has not yet been reported in HNSCC, well-designed studies are warranted in future, especially regarding HPV- or EBV- driven HNSCCs that harbor a restively high tendency of distant metastasis.

### 8.2. Volume and Number of Sites to Irradiate

Accumulating evidence has supported the feasibility of multi-site irradiation in a metastatic setting. As was illustrated in detail by Chang et al. [97], multi-site RT can overcome the inter-metastatic heterogeneity of tumor tissue by releasing more diversified tumor antigens into circulation, which has been suggested to trigger a distant anti-tumor response in preclinical models [98]. Meanwhile, it may guarantee the immunomodulatory effect of RT in more metastatic sites. Moreover, multi-site RT maximally restores the vasculature of metastatic lesions to enable the penetration of immune cells. However, adopting multi-site RT in conjunction with ICIs also raised some questions with time and economic considerations. Due to the lack of data in HNSCC including HPV-positive cancers, whether the combination is related to increased toxicities will also have to be answered in future studies. 

Partial irradiation of metastases has also been proposed, based on preclinical studies that showed non-inferiority of irradiation to 50% of the volume of the tumor to that of the full volume. In the phase I NRG-BR001 trial that enrolled 79 metastatic patients including four with HNSCC, Luke et al. reported that partial SBRT to > 65 cm^3^ metastases followed by pembrolizumab resulted in a local control comparable to that targeting the entire metastatic volume [99]. This result might extrapolate to HPV-associated HNSCC in clinical practice, though prospective trials would be difficult due to a small sample size. 

Elective nodal irradiation (ENI), usually known as the prophylactic delivery of a radiation dose to the clinically uninvolved but at-risk nodal areas, targets subclinical metastases in draining lymph nodes (DLN) and is routinely used in HNSCC in the definitive setting, including HPV-associated HNSCC. However, with the growing importance of DLN as a major location of T-cell priming, larger-field ENI might hinder this process by directly killing CD8+ T-cells. In fact, ENI has failed to improve outcomes in multiple malignancies including esophageal squamous cell carcinoma [100], early-stage NSCLC [101], prostate cancer [102], etc. A preclinical model even found ENI with worse outcomes when combined with SBRT and ICIs, due to an adverse effect on adaptive immune responses by altering chemokine expression and trafficking of CD8+ T-cells [103]. This is in line with the failure of the JAVELIN head and neck 100 trial where ENI was frequently given and the success of the NIRT-HNC trial that used SBRT to gross tumor volume only, as were above mentioned in this review. As more interest are now focusing on definitive radioimmunotherapy in HNSCC, especially HPV-associated diseases, future trials will be encouraged on reduced-volume irradiation and avoidance of unnecessary ENI to maximize the therapeutic effects.

### 8.3. Timing of Immunotherapy to Radiotherapy

Timing of ICIs to irradiation is of essential importance to exert a synergistic efficacy. According to Dovedi et al., effective anti-tumor response and long-term tumor control can be acquired only when PD-L1 blockade was administered concurrently with RT [104]. Retrospectively data suggested that ICIs concurrent with RT conferred advantages in ORR and OS over the sequential scheduling [105,106]. Preliminary results from a phase I COSINR study also favored concurrent use of nivolumab and ipilimumab with multi-site RT, given a higher cytoreduction of non-irradiated metastases (abscopal response) demonstrated by deep learning [107]. However, a phase II randomized trial (NCT02777385) in LA-HNSCC reported at the ASCO 2022 annual meeting that sequential pembrolizumab added to chemoradiotherapy yielded numerically superior 1- and 2-year PFS when compared with the concurrent use, thus proposing sequential scheduling as the priority option [108]. It remains to be elucidated whether the best sequencing differs with metastatic/definitive setting, fractionation dose of RT, tumor species and types of immunotherapy agents.

### 8.4. Synergistic Agents with Radioimmunotherapy

Theoretically, any agent that acts against the intrinsic resistance of HNSCC might synergize with radioimmunotherapy to expand the therapeutic ratio. Based on the potential mechanisms of resistance, the combinational strategy could involve several aspects: (1) chemotherapy [109] that induces tumor debulking and immunogenic cell death, such as nabpaclitaxel, carboplatin, gemcitabine and 5-Fu; (2) molecular therapy [110] that targets aberrant cellular signal pathways such as EGFR pathway (cetuximab), angiogenesis-hypoxia pathway (bevacizumab, lenvatinib), PI3K/AKT/mTOR pathway (rapamycin, everolimus), cell cycle pathway (palbociclib), DNA repair pathway (olaparib) and so on; (3) blockade of different immune checkpoints [111] such as TIM-3, LAG-3, TIGIT, VISTA and so on; (4) immunotherapy that targets cytokines (IL-2, IFN-α) and soluble factors (GM-CSF, anti-TGF-β antibodies) to promote anti-cancer immunity [112]; (5) adoptive cell therapy (CAR-T, CAR-NK) [113]. However, little is known about the safety profile and appropriate indications for this triplet regimen till now, and more evidence will be found in future trials. 

Of note, the unique biological features of HPV-associated HNSCC could provide insight into disease-specific strategies. For instance, DNA immunotherapy targeting E6/E7 stimulated potent anti-tumor immune responses in a phase Ib/II trial [114], whether this approach could provide additional benefit to radioimmunotherapy will need to be investigated. Besides, Treg depletion offers a hopeful solution in HPV-associated HNSCC, given the fact that Treg enrichment constitutes a major cause of immunosuppression in this entity and preclinical evidence showing that tumor got eradicated in radiotherapy only when preconditioned with Treg depletion [115].

## 9. Conclusions

In summary, current evidence indicates that HPV-associated HNSCC has higher sensitivity to RT and immunotherapy. Given the synergetic effect of RT and ICIs, HPV-associated HNSCC may represent a favorable candidate for radioimmunotherapy due to its special biological, genomic and immunologic landscape. Preliminary studies, although rare, have indicated the tolerance and efficacy of radioimmunotherapy in HPV-associated HNSCC. Ongoing trials will further provide valuable insight into the potential of radioimmunotherapy in triggering abscopal effect in the metastatic setting and facilitating personalized treatment in the definitive setting, as well as the safety profiles in HPV-associated HNSCC. There are open questions regarding the optimal RT scheduling, volume, and sites to irradiate and the combinational agents to elicit the synergetic effect of RT and immunotherapy, future investigations in these fields should be encouraged. 

## Figures and Tables

**Table 1:** Prospective clinical trials involving radiotherapy and ICIs in recurrent/metastatic HPV-positive HNSCC.

ClinicalTrials.gov Identifier	Title	Phase	Patient Population	Interventional Arms	RT Prescription	Timing of ICI to RT
NCT02684253	Screening Trial of Nivolumab With Image Guided, Stereotactic Body Radiotherapy (SBRT) Versus Nivolumab Alone in Patients With Metastatic Head and Neck Squamous Cell Carcinoma (HNSCC)	II	Metastatic HNSCC, including HPV+	SBRT + nivolumab	27 Gy in 3 fractions, single lesion	Concurrent + maintenance
NCT04830267	The Efficacy of Camrelizumab Plus Stereotactic Body Radiotherapy in R/M HNSCC	II	Recurrent/metastatic HNSCC, including HPV+	SBRT + Camrelizumab	27 Gy in 3 fractions, single lesion	Neoadjuvant + concurrent + maintenance
NCT04576091	Testing the Addition of an Anti-cancer Drug, BAY 1895344, With Radiation Therapy to the Usual Pembrolizumab Treatment for Recurrent Head and Neck Cancer	I	Recurrent, unresectable HNSCC, including HPV+	SBRT + pembrolizumab + elimusertib	Unspecified dose in 3 factions	Neoadjuvant + concurrent

Abbreviations: ICI = immune checkpoint inhibitors; HPV = human papillomavirus; HNSCC = head and neck squamous cell carcinoma; RT = radiotherapy; SBRT = stereotactic body radiotherapy.

**Table 2:** Prospective clinical trials involving radiotherapy and ICIs in loco-regional HPV-positive HNSCC.

ClinicalTrials.gov Identifier	Title	Phase	Patient Population	Interventional Arms	RT Prescription	Timing of ICI to RT
NCT03811015	EA3161: Testing Immunotherapy Versus Observation in Patients With HPV Throat Cancer	II/III	Intermediate risk LA-OPC, HPV+	CRT + nivolumab	70 Gy in 35 fractions	Adjuvant
NCT03452137	IMvoke010: A Study of Atezolizumab (Anti-Pd-L1 Antibody) as Adjuvant Therapy After Definitive Local Therapy in Patients With High-Risk Locally Advanced Squamous Cell Carcinoma of the Head and Neck	III	High-risk LA-HNSCC, including HPV+	Surgery or RT + atezolizumab	NA	Adjuvant
NCT02952586	JAVELIN Head and Neck 100: Study To Compare Avelumab In Combination With Standard of Care Chemoradiotherapy (SoC CRT) Versus SoC CRT for Definitive Treatment In Patients With Locally Advanced Squamous Cell Carcinoma Of The Head And Neck (JAVELIN HEAD AND NECK 100)	III	LA-HNSCC, including HPV+	CRT/avelumab + avelumab	70 Gy in 35 fractions	Concurrent + adjuvant
NCT02586207	Pembrolizumab in Combination With CRT for LA-SCCHN	Ib	LA-HNSCC, including HPV+	CRT/pembrolizumab + pembrolizumab	70 Gy in 35 fractions	Concurrent + adjuvant
NCT03040999	Study of Pembrolizumab (MK-3475) or Placebo With Chemoradiation in Participants With Locally Advanced Head and Neck Squamous Cell Carcinoma (MK-3475-412/KEYNOTE-412)	III	LA-HNSCC, including HPV+	CRT/pembrolizumab + pembrolizumab	70 Gy in 35 fractions	Concurrent + adjuvant
NCT02819752	PEACH: PEmbrolizumab Combined With Chemoradiotherapy in Squamous Cell Carcinoma of the Head and Neck	I	LA-HNSCC, including HPV+	CRT/pembrolizumab + pembrolizumab	NA	Concurrent + adjuvant
NCT04369937	HPV-16 Vaccination and Pembrolizumab Plus Cisplatin for “Intermediate Risk” HPV-16-associated Head and Neck Squamous Cell Carcinoma	II	Intermediate risk LA-HNSCC, HPV 16+	CRT/pembrolizumab /ISA101b	70 Gy in 35 fractions	Concurrent
NCT03838263	IMMUNEBOOST: Feasibility and Tolerance of Nivolumab Neoadjuvant Immunotherapy in High Risk HPV Driven Oropharynx Cancer	II	High risk OPC, HPV+	Nivolumab + CRT	70 Gy in 35 fractions	Neoadjuvant
NCT04116047	CompARE: Escalating Treatment of Intermediate and High-risk Oropharyngeal Cancer (OPC)	III	Intermediate-high risk OPC, including HPV+	Durvalumab + CRT + Durvalumab	70 Gy in 35 fractions	Neoadjuvant + adjuvant
NCT05366166	Pembrolizumab Plus Olaparib in LA-HNSCC	II	High-risk LA-HNSCC, including HPV+	Pembrolizumab/olaparib + CRT + pembrolizumab/olaparib	70 Gy in 35 fractions	Neoadjuvant + adjuvant
NCT03829722	Radiotherapy, Carboplatin/Paclitaxel and Nivolumab for High Risk HPV-related Head and Neck Cancer	II	High-risk LA-HNSCC, HPV+	Nivolumab + RT/carboplatin/paclitaxel/nivolumab + nivolumab	70 Gy in 35 fractions	Neoadjuvant + concurrent + adjuvant
NCT02707588	PembroRad: Tolerance and Efficacy of Pembrolizumab or Cetuximab Combined With RT in Patients With Locally Advanced HNSCC	II	High-risk HNSCC, including HPV+	RT/pembrolizumab	69.96 Gy in 33 fractions	Concurrent
NCT03383094	KEYCHAIN: Chemoradiation vs. Immunotherapy and Radiation for Head and Neck Cancer	II	Intermediate-high risk HNSCC, p16+	RT/pembrolizumab + pembrolizumab	70 Gy in 33–35 fractions	Concurrent + adjuvant
NCT03258554	NRG-HN004: Radiation Therapy With Durvalumab or Cetuximab in Treating Patients With Locoregionally Advanced Head and Neck Cancer Who Cannot Take Cisplatin	II/III	LA-HNSCC, including HPV+	RT/Durvalumab + Durvalumab	70 Gy in 35 fractions	Concurrent + adjuvant
NCT03952585	NRG-HN005: De-intensified Radiation Therapy With Chemotherapy (Cisplatin) or Immunotherapy (Nivolumab) in Treating Patients With Early-Stage, HPV-Positive, Non-Smoking Associated Oropharyngeal Cancer	II/III	Early-stage, non-smoking OPC, p16+	RT/nivolumab	60 Gy in 30 fractions	Concurrent
NCT03623646	CITHARE: Cisplatin or ImmunoTHerapy in Association With Definitive Radiotherapy in HPV-related oropharyngEal Squamous Cell Carcinoma: a Randomized Phase II Trial	II	Early-stage OPC, p16+	RT/Durvalumab	70 Gy in 35 fractions	Concurrent
NCT03410615	CCTG HN.9: Cisplatin + Radiotherapy vs. Durvalumab + Radiotherapy Followed by Durvalumab vs. Durvalumab + Radiotherapy Followed by Tremelimumab + Durvalumab in Intermediate-Risk HPV-Positive Oropharyngeal SCC	II	Intermediate risk LA-OPC, HPV-positive	Radiation/durvalumab + durvalumab ± tremelimumab	70 Gy in 35 fractions	Concurrent + adjuvant
NCT02999087	REACH: Randomized Trial of Avelumab-cetuximab-radiotherapy Versus SOCs in LA SCCHN (REACH)	III	LA-HNSCC, including HPV+	RT/cetuximab/avelumab + avelumab	69.96 Gy in 33 fractions	Concurrent + adjuvant
NCT03799445	Ipilimumab, Nivolumab, and Radiation Therapy in Treating Patients With HPV Positive Advanced Oropharyngeal Squamous Cell Carcinoma	II	Low-intermediate volume LA-OPC, HPV+	Nivolumab/ipilimumab + RT/nivolumab/ipilimumab	NA	Neoadjuvant + concurrent
NCT03162731	Nivolumab, Ipilimumab, and Radiation Therapy in Treating Patients With Stage III-IVB Head and Neck Cancer	I	LA-HNSCC, including HPV+	Nivolumab/ipilimumab + RT/nivolumab/ipilimumab + nivolumab/ipilimumab	70 Gy in 35 fractions	Neoadjuvant + concurrent + adjuvant
NCT03715946	Adjuvant De-Escalated Radiation + Adjuvant Nivolumab for Intermediate-High Risk P16+ Oropharynx Cancer	II	Intermediate-high risk OPC, p16+	TOS + RT/nivolumab + nivolumab	45 or 50 Gy in 25 daily fractions	Concurrent + adjuvant
NCT04867330	IChoice-02: Toripalimab Based Induction Chemotherapy Followed by De-escalation Protocols in HPV-related OPSCC	II	LA-OPC, HPV+	Toripalimab/docetaxel /cisplatin + RT	60 Gy in 30 fractions	Neoadjuvant
NCT03107182	OPTIMA-II: Chemotherapy and Locoregional Therapy Trial (Surgery or Radiation) for Patients With Head and Neck Cancer	II	LA-HNSCC, HPV+	Nivolumab/nab-paclitaxel/carboplatin + risk-adapted TORS or RT or CRT	Response-stratified to: arm 1: RT with 50 Gy in 25 fractions; arm 2: CRT with cisplatin to 50 Gy, or TFHX to 45 Gy; arm 3: CRT with cisplatin to 70 Gy, or TFHX to 75 Gy.	Neoadjuvant + adjuvant
NCT03426657	CheckRad-CD8: Radiotherapy With Double Checkpoint Blockade of Locally Advanced HNSCC	NA	LA-HNSCC, including HPV+	Docetaxel/cisplatin/durvalumab /tremelimumab + RT/durvalumab /tremelimumab + durvalumab	70 Gy in 35 fractions	Neoadjuvant + concurrent + adjuvant
NCT03247712	NIRT-HNC: Neoadjuvant Immunoradiotherapy in Head & Neck Cancer	I/II	HNSCC, HPV+	SBRT/nivolumab + surgery + nivolumab	40 Gy in 5 fractions or 24 Gy in 3 fractions	Concurrent + adjuvant
NCT03618134	Stereotactic Body Radiation Therapy and Durvalumab With or Without Tremelimumab Before Surgery in Treating Participants With Human Papillomavirus Positive Oropharyngeal Squamous Cell Cancer	I/II	HNSCC, HPV+	SBRT/(durvalumab ± tremelimumab) + surgery + durvalumab	NA	Concurrent + adjuvant

Abbreviations: ICI = immune checkpoint inhibitors; HPV = human papillomavirus; HNSCC = head and neck squamous cell carcinoma; LA = locoregional advanced; OPC = oropharyngeal carcinoma; CRT = chemoradiotherapy; RT = radiotherapy; SBRT = stereotactic body radiotherapy; TOS = transoral surgery; TORS = transoral robotic surgery; SoC = standard of care; TFHX = paclitaxel, 5-FU, hydroxyurea, dexamethasone, famotidine, and diphenhydramine; NA = not available.

## Data Availability

Not applicable.
